# Human Wharton’s Jelly Mesenchymal Stromal Cell-Derived Small Extracellular Vesicles Drive Oligodendroglial Maturation by Restraining MAPK/ERK and Notch Signaling Pathways

**DOI:** 10.3389/fcell.2021.622539

**Published:** 2021-03-23

**Authors:** Marianne S. Joerger-Messerli, Gierin Thomi, Valérie Haesler, Irene Keller, Patricia Renz, Daniel V. Surbek, Andreina Schoeberlein

**Affiliations:** ^1^Department of Obstetrics and Feto-maternal Medicine, Inselspital, Bern University Hospital, University of Bern, Bern, Switzerland; ^2^Department for BioMedical Research (DBMR), University of Bern, Bern, Switzerland; ^3^Graduate School for Cellular and Biomedical Sciences, University of Bern, Bern, Switzerland; ^4^Swiss Institute of Bioinformatics, University of Bern, Bern, Switzerland

**Keywords:** mesenchymal stromal cells, Wharton’s jelly, small extracellular vesicles, premature white matter injury, oligodendroglial maturation, ERK, Notch, microRNA

## Abstract

Peripartum cerebral hypoxia and ischemia, and intrauterine infection and inflammation, are detrimental for the precursor cells of the myelin-forming oligodendrocytes in the prematurely newborn, potentially leading to white matter injury (WMI) with long-term neurodevelopmental sequelae. Previous data show that hypomyelination observed in WMI is caused by arrested oligodendroglial maturation rather than oligodendrocyte-specific cell death. In a rat model of premature WMI, we have recently shown that small extracellular vesicles (sEV) derived from Wharton’s jelly mesenchymal stromal cells (WJ-MSC) protect from myelination deficits. Thus, we hypothesized that sEV derived from WJ-MSC directly promote oligodendroglial maturation in oligodendrocyte precursor cells. To test this assumption, sEV were isolated from culture supernatants of human WJ-MSC by ultracentrifugation and co-cultured with the human immortalized oligodendrocyte precursor cell line MO3.13. As many regulatory functions in WMI have been ascribed to microRNA (miR) and as sEV are carriers of functional miR which can be delivered to target cells, we characterized and quantified the miR content of WJ-MSC-derived sEV by next-generation sequencing. We found that WJ-MSC-derived sEV co-localized with MO3.13 cells within 4 h. After 5 days of co-culture, the expression of myelin basic protein (MBP), a marker for mature oligodendrocytes, was significantly increased, while the oligodendrocyte precursor marker platelet-derived growth factor alpha (PDGFRα) was decreased. Notch and MAPK/ERK pathways known to inhibit oligodendrocyte maturation and differentiation were significantly reduced. The pathway enrichment analysis showed that the miR present in WJ-MSC-derived sEV target genes having key roles in the MAPK pathway. Our data strongly suggest that sEV from WJ-MSC directly drive the maturation of oligodendrocyte precursor cells by repressing Notch and MAPK/ERK signaling.

## Introduction

White matter injury (WMI) is the most common cerebral abnormality in premature neonates. Thanks to improved peripartum care in preterm birth, the number of newborns affected by the cystic form of WMI, called cystic periventricular leukomalacia (PVL), has become very low nowadays (Hamrick et al., 2004). However, the non-cystic and mostly diffuse type of cerebral WMI remains predominant in very preterm-born infants (Inder et al., 2003; Hamrick et al., 2004; Horsch et al., 2007). The frequency of preterm WMI, counting both cystic and non-cystic, is 39.6% in infants born before gestational week 28, 27.4% before week 32, and 7.3% before week 37 (Romero-Guzman and Lopez-Munoz, 2017). Long-term neurological consequences are typically cerebral palsy in cystic WMI and cognitive deficits and impairments in attention, behavior, or socialization in diffuse cerebral WMI (Agut et al., 2020).

The pathophysiology of premature WMI is complex. However, it is generally accepted to relate to both fetal cerebral hypoxia and ischemia and intrauterine infection and inflammation causing downstream excitotoxicity and oxidative stress, which are harmful to the maturing myelin-forming oligodendrocyte lineage (Khwaja and Volpe, 2008; Volpe, 2008). Unfortunately, today no effective therapy for preterm WMI is available.

In preclinical models of premature WMI, mesenchymal stromal cell (MSC)-based therapy turned out to be an effective treatment, in which the beneficial effects rely to a great extent on secreted extracellular vesicles (EV) (Katsuda et al., 2013; Lener et al., 2015). According to their size, EV can be distinguished between small EV (sEV) (50–150 nm in diameter), also known as exosomes, and microvesicles (≤1,000 nm in diameter) (Théry et al., 2018; Rohde et al., 2019). EV can transfer at least parts of the biological properties of their mother cells by releasing their cargo, consisting of proteins, lipids and nucleic acids, into their target cell (van Niel et al., 2018). The use of EV as biological treatment has several advantages over classical cell-based therapy. EV are not viable, making it less complex to store them and to standardize the applied dose together with the biological activity. Furthermore, they are not tumorigenic and less immunogenic than proliferating cells.

Pre-clinically, MSC-derived EV have been proven to display neuroprotective and –regenerative effects comparable to their cells of origin (Vizoso et al., 2017). For instance, in ovine fetuses, MSC-derived EV reduced the hypo-myelination and improved the brain functions in a model of perinatal brain injury (Ophelders et al., 2016). In a rat model of premature brain injury induced by inflammation, the treatment with MSC-derived EV restored white matter microstructure and myelination (Drommelschmidt et al., 2017). We have recently shown that intranasal administration of sEV derived from MSC isolated from the umbilical cord connective tissue, called Wharton’s jelly (WJ), reduced WMI in a preclinical rat model of premature brain injury (Thomi et al., 2019a). In accordance with others, we further provided strong pieces of evidence that the WMI in premature brains is caused by disturbed oligodendrocyte maturation rather than the cell death of the oligodendrocyte lineage (Buser et al., 2012; Oppliger et al., 2016; van Tilborg et al., 2018; Thomi et al., 2019a).

A crucial role in neurodevelopment and -regeneration has been ascribed to microRNAs (miR), which–besides other functional molecules–can be delivered by sEV to their recipient cells and exert their task. MicroRNAs have first been described in 1993 by Lee and coworkers in *C. elegans* (Lee et al., 1993). They are short non-coding RNAs, which are highly conserved across species. The key function of miR is posttranscriptional gene regulation leading to the cleavage, translational repression or deadenylation of the target genes (Winter et al., 2009). Thereby, the miR bind to the 3′ UTR of the repressing mRNA forming the so-called RNA-induced silencing (RISC) complex. Because of their crucial role in gene silencing, we hypothesize that WJ-MSC-derived sEV contain miR that interfere with signaling pathways involved in WMI.

Based on these findings, we aimed to explore the neuroregenerative potential of WJ-MSC-derived sEV in WMI by analyzing their effect on the maturation of oligodendroglial progenitor (OPC)-like cells and the characterization and quantification of the miR cargo of sEV.

## Materials and Methods

### Isolation of sEV Derived From Human Wharton’s Jelly-Derived Mesenchymal Stromal Cells (WJ-MSC)

After cesarean section, umbilical cords from healthy term deliveries (gestational age ≥ 37 weeks) were collected from patients after obtaining informed consent. The study was approved by the Ethics Committee of the Canton of Bern (reference numbers: KEK BE 090_07 and KEK BE 178_03). Human WJ-MSC were isolated from the umbilical cord connective tissue, the so-called Wharton’s jelly, via enzymatic digestion as previously described (Joerger-Messerli et al., 2018). The cells were expanded in Dulbecco’s modified Eagle’s medium (DMEM)/F12 supplemented with 10% fetal bovine serum (FBS), 2 mmol/L GlutaMAX^TM^, 100 units/mL penicillin and 100 μg/mL streptomycin (Thermo Fisher Scientific, Waltham, MA, United States). The WJ-MSC’s identity is regularly confirmed, as we have previously published (Messerli et al., 2013; Thomi et al., 2019b). At passage 4–6, WJ-MSC were split into eight T150 cm^2^ cell culture flasks and cultured until they reached 80% confluency. Then, the cells were washed twice with PBS, and the medium was replaced with serum-free DMEM/F12, supplemented with 2 mmol/L GlutaMAX^TM^, 100 units/mL penicillin, and 100 μg/mL streptomycin. After 36 h of incubation, culture supernatants were collected and sEV were isolated by serial centrifugations according to the protocol of Théry et al. (2006). The pelleted sEV were retrieved in PBS and stored at −80°C. The sEV were characterized according to their morphology, size, and endosomal marker expression, as we have recently published (Thomi et al., 2019b).

### Culture of MO3.13 Cells With WJ-MSC-Derived sEV

The human oligodendrocytic hybrid cell line MO3.13 was expanded in Dulbecco’s Modified Eagle’s Medium (DMEM) supplemented with 10% FBS, 2 mmol/L GlutaMAX^TM^, 100 units/mL penicillin, and 100 mg/mL streptomycin. The cells were detached by adding 0.25% trypsin/EDTA.

For the culture with WJ-MSC-derived sEV, MO3.13 cells were seeded at a density of 3,500 cells/cm^2^. MO3.13 cells were let to adhere overnight. The next day, sEV (0.5 μg/mL) were added and cultured for 5 days. On day 3, the medium was changed, and fresh sEV (0.5 μg/mL) were added. As a control, the cells were cultured without sEV.

To analyze the interaction between sEV and MO3.13, sEV were stained with 2 × 10^–6^ M PKH26 (Sigma-Aldrich, St. Louis, MO, United States) as we have recently described (Thomi et al., 2019b). MO3.13 were seeded at a density of 2,500 cells/cm^2^ in Nunc^®^, Lab-Tek^®^, 2-chamber slides (Sigma-Aldrich). To avoid overgrowth and properly detect interactions between single MO3.13 and sEV, the cells were seeded less dense than for the experiments described above. The next day, PKH26-labeled sEV were co-cultured with MO3.13 for 4 h.

### Immunocytochemistry

After co-culture of MO3.13 cells and sEV, the cells were fixed with 4% paraformaldehyde and blocked with 1% bovine serum albumin (BSA; Sigma-Aldrich) and 0.25% Triton X-100 (Sigma-Aldrich) in PBS for 1 h at room temperature. MO3.13 were stained overnight at 4°C with antibodies against the following proteins: β-tubulin (1:200, ab6046, Abcam, Cambridge, United Kingdom), galactocerebrosidase (GalC, 1:500, G9152, Sigma-Aldrich), 2′,3′-cyclic-nucleotide 3′-phosphodiesterase (CNPase, 1:100, C5922, Sigma-Aldrich), myelin basic protein (MBP, 1:200, ab40390, Abcam), and myelin-associated glycoprotein (MAG, 1:400, ab89780, Abcam). For the detection, anti-rabbit IgG Alexa Fluor 488 antibody (1:200, Thermo Fisher Scientific) and anti-mouse IgG Alex Fluor 594 antibody (1:200, Thermo Fisher Scientific) were used and incubated for 1 h at room temperature. 4′,6-diamidino-2′-phenylindole-dihydrochloride (DAPI; Sigma-Aldrich) was used to counterstain the nuclei. Images were either acquired on a DM6000 B microscope (Leica Microsystems, Wetzlar, Germany), or on a laser scanning microscope (Carl Zeiss LSM 710, Oberkochen, Germany) and processed using the Zeiss ZEN lite imaging software version 3.3 (Carl Zeiss). Randomly selected fields of vision (*n* = 14) were chosen to calculate the percentage of PKH26-positive MO3.13 cells relative to the number of DAPI-positive nuclei.

### RNA and Protein Extraction

RNA and protein of WJ-MSC were isolated by the use of the QIAshredder and the Allprep DNA/RNA/Protein Mini Kit according to the manufacturers’ protocol (Qiagen, Hilden, Germany). Total Exosome RNA and Protein Isolation Kit (Thermo Fisher Scientific) was used to extract the total RNA of WJ-MSC-derived sEV according to the manufacturers’ instructions. RNA concentration was measured by NanoVue Plus^TM^ spectrophotometer (Biochrom, Holliston, MA, United States). The Bicinchoninic Acid Protein Assay Kit (Sigma-Aldrich) was used for the determination of the total protein concentration of WJ-MSC.

### Reverse Transcription and Real-Time Polymerase Chain Reaction (PCR)

The reverse transcription of mRNA was done with up to 3 μg RNA using the SuperScript VI First-Strand Synthesis System (Thermo Fisher Scientific). Gene expression was measured by real-time RT-qPCR using the primercon assays listed in Table 1. The following PCR cycling program was run on a QuantStudio ^TM^7 Flex Real-Time PCR System (Thermo Fisher Scientific): 2 min at 50°C, 10 min at 95°C, followed by 45 cycles of 15 s at 95°C and 1 min at 60°C. The housekeeping gene glyceraldehyde-3-phosphate dehydrogenase was used as an endogenous control. Analysis of all real-time PCR reactions was done using the QuantStudio^TM^ 7 Flex Real-Time PCR System Software. Data were expressed as fold change relative to commercial total human RNA (Thermo Fisher Scientific).

**TABLE 1:** Primers and probes or gene expression assays used for real-time RT-qPCR.

**Gene**	**Description**	**Assay ID/Primers and probe sequences *Homo sapiens***
*HES5*	Hes family bHLH transcription factor 5	Hs01387463_g1
*MAPK1*	Mitogen-activated protein kinase 1	Hs01046830_m1
*MBP*	Myelin basic protein	F: 5′-ACTATCTCTTCCTCCCAGCTTAAAAA-3′
		R: 5′-TCCGACTATAAATCGGCTCACA-3′
		P: 5′-TGGGCATCGACTCCCTTGAATCCC-3′
*NOTCH1*	Notch homolog 1	F: 5′-TGCATGATGCCTACATTTCAAGA-3′
		R: 5′-TTCAGTATTATGTAGTTGTTCGTTGGTTATAC-3′
		P: 5′-TGGTTCTGGAGGGACC-3′
*NOTCH2*	Notch homolog 2	Hs01050702_m1
*NRAS*	Neuroblastoma RAS viral oncogene homolog	Hs00180035_m1
*PDGFR*α	Platelet-derived growth factor receptor alpha	Hs00998018_m1

**F, Forward Primer; R, Reverse Primer; P, Probe.**

### Western Blot Analysis

Extracted proteins were separated by sodiumdodecylsulfate–polyacrylamide gel electrophoresis (SDS-PAGE) on a 4–20% gradient gel (Bio-Rad, Hercules, CA, United States), transferred to nitrocellulose membranes (Thermo Fisher Scientific), and blocked either with 5% non-fat dry milk (NFDM) or 5% bovine serum albumin (BSA, Sigma-Aldrich) dissolved in Tris-buffered saline (TBS). The following proteins were analyzed: Cleaved Notch1 (4147, Cell Signaling Technology, Inc., Danvers, MA, United States), platelet-derived growth factor receptor-alpha (PDGFRα, ab61219, Abcam), myelin basic protein (Mbp, AB980, Thermo Fisher Scientific), proliferating-cell-nuclear-antigen (PCNA, 2586, Cell Signaling Technology), Erk1/2 (9102, Cell Signaling Technology), Phospho-Erk1/2 (9101, Cell Signaling Technology), β-Tubulin (ab6046, Abcam). Horseradish peroxidase-coupled donkey anti-rabbit or sheep anti-mouse antibodies (Cytiva, Marlborough, MA, United States) were used as secondary antibodies. Binding was detected using the chemiluminescent Amersham ECL Prime western Blotting Detection Reagent (Cytiva) on a Chemidoc XRS + system (Bio-Rad). Pixel summation of individual bands was performed with ImageJ Software (NIH, Bethesda, MD, United States).

### RNA Sequencing and Bioinformatics Processing

Genome-wide profiling of paired WJ-MSC and WJ-MSC-derived sEV from the same individual (*n* = 6) was done by preparing small RNA libraries, and sequencing on a Illumina HiSeq 4000 sequencing system (Illumina, San Diego, CA, United States) by Fasteris SA (Plan-les-Ouates, Switzerland). We used MirDeep2 v. 0.1.2 (Friedlander et al., 2012) to map the reads to the human reference genome (GRCh38) and count the number of reads per known miRNA (miRBase v. 22).

### Functional Analysis of sEV-Derived miR

A volcano plot presenting up- and down-regulated miR in sEV relative to their parental cells was done using *R* (R Core Team, 2020). To create heatmaps showing the expression of the most abundant miR (mean normalized counts >10,000) in sEV relative to WJ-MSC and vice versa, we used the freely available web server Heatmapper (Babicki et al., 2016). The FunRich functional enrichment analysis tool version 3.1.3 was used to create a Venn diagram (Pathan et al., 2015; Pathan et al., 2017).

Kyoto Encyclopedia of Genes and Genomes (KEGG) pathway enrichment analysis of miR in sEV with ≥10,000 mean normalized counts was performed using DIANA-miRPath 3.0 web-server (Vlachos et al., 2015). The rich factor was calculated as the ratio of the number of targeted genes divided by the number of all the genes in each pathway. The *Q*-value is the False Discovery Rate (FDR)-adjusted *p*-value. The results were shown as a scatter plot using *R* (R Core Team, 2020). To illustrate a miR-mRNA network, Cytoscape software version 3.7.1 was used (Shannon et al., 2003).

### Statistical Analysis

The statistical analysis of the real-time PCR and western blot results have been done using GraphPad Prism version 7.00 for Windows (Graphpad Software, San Diego, California United States, www.graphpad.com). To compare gene and protein expression in untreated and sEV-treated MO3.13 cells paired *t*-test was done. *p* < 0.05 was considered statistically significant.

To test for significant differences in miR expression between WJ-MSC-derived EV and WJ-MSC, DESeq2 v. 1.22.2 in R v. 3.5.2 (Love et al., 2014) was used. *p*-values were FDR-adjusted utilizing the procedure of Benjamini-Hochberg (*p*-adj). *p*-adj < 0.05 was considered statistically significant.

## Results

### sEV Co-localize With MO3.13 Cells

**FIGURE 1:**
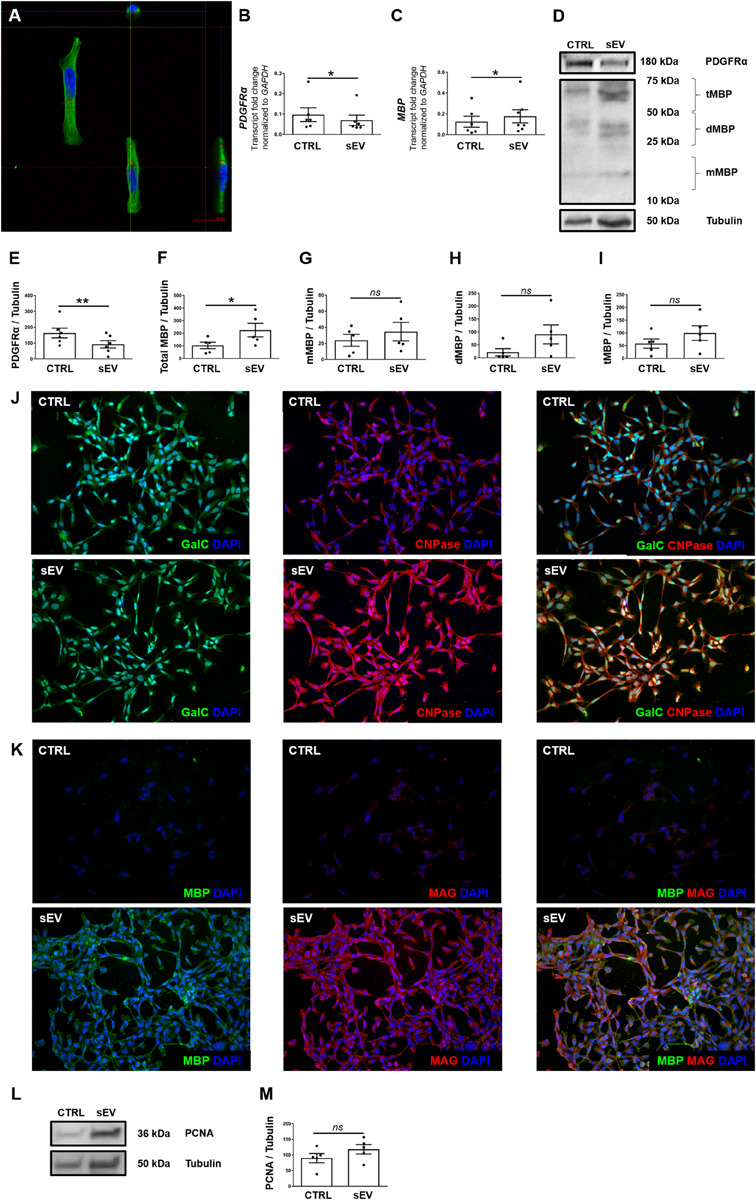
WJ-MSC-derived sEV co-localize with MO3.13 cells and drive their maturation. **(A)** Representative confocal Z-stack section after co-culture of sEV with MO3.13 cells for 4 h. To label sEV, the fluorescent membrane dye PKH26 (red) was used. MO3.13 cells were stained with β-tubulin (green). Their nuclei were counterstained with 4′,6-diamidino-2′-phenylindole-dihydrochloride (DAPI) (blue). **(B,C)** Transcription of *PDGFR*α (*n* = 6) and *MBP* (*n* = 6) by MO3.13 cells either left untreated or co-incubated with 0.5 μg/mL sEV for 5 days. **(D)** Representative western blots of PDGFRα and MBP expression by MO3.13 cells either left untreated or co-cultured with 5 μg/mL sEV for 5 days. **(E)** Quantification of PDGFRα protein expression by MO3.13 cells after 5 days (*n* = 6) **(F–I)** Quantification of MBP protein expression by MO3.13 cells after 5 days (*n* = 5). **(J,K)** Representative fluorescence microscopy images of either untreated or sEV-treated MO3.13 cells after 5 days for the expression of GalC, CNPase, MBP, and MAG. Their nuclei were counterstained with DAPI. **(L)** Representative western blot of PCNA expression by MO3.13 cells either left untreated or co-cultured with 5 μg/mL sEV for 5 days. **(M)** Quantification of PCNA protein expression by MO3.13 cells after 5 days (*n* = 5). Data are presented as mean ± SEM. *p < 0.05, **p < 0.01, *ns*, non-significant.

Physical contact with the target cell is essential for sEV to have a potential therapeutic effect. Therefore, PKH26-labeled sEV were co-cultured with the immortalized oligodendrocyte precursor cell line MO3.13. After 4 h of co-culture, confocal microscopy revealed co-localization of PKH26-labeled sEV and MO3.13 stained for β-tubulin (Figure 1A). sEV mostly formed aggregates and were located in the perinuclear region, indicating their complete internalization. The percentage of PKH26-positive MO3.13 cells was 22.97 ± 20.69.

### sEV Reduce PDGFRα and Induce MBP Expression in MO3.13 Cells

We measured the expression of platelet-derived growth factor alpha (PDGFRα) and myelin basic protein (MBP) to assess the effect of WJ-MSC-derived sEV on the maturation of MO3.13 cells. After 5 days of co-culture with sEV, the transcription of *PDGFR*α was reduced (*p* = 0.0339), whereas the gene expression of *MBP* was increased in MO3.13 (*p* = 0.022) compared to untreated MO3.13 cells (Figures 1B,C). The protein levels of PDGFRα in MO3.13 cells were decreased as well after 5 days of co-culture with sEV related to untreated cells (*p* = 0.0045) (Figures 1D,E). Western blot analysis of MBP revealed several signals at distinct sizes (Figure 1D). The size of the classic MBP isoforms ranges from 14 to 21.5 kDa. Western blot analysis showed a faint signal at a size of 20 kDa representing monomeric MBP isoforms (mMBP). The MBP signals between 25 and 50 kDa, and 50 and 75 kDa, respectively, are most likely representing dimeric MBP (dMBP) and trimeric (tMBP), as MBP tends to self-associate (Smith, 1982). The expression of all MBP signals together was significantly increased in MO3.13 cells upon 5 days of co-culture with sEV compared to untreated cells (*p* = 0.022) (Figures 1F–I). Immunofluorescent stainings showed that untreated MO3.13 expressed GalC and CNPase. The co-culture of MO3.13 cells with sEV for 5 days intensified the signal of CNPase (Figure 1J). In contrast, there was nearly no expression of MBP and MAG) in untreated MO3.13 cells, which was clearly increased upon sEV treatment for 5 days (Figure 1K). Furthermore, as the decreasing proliferation of cells is a further sign of maturation, we measured the proliferating-cell-nuclear-antigen (PCNA) protein expression in untreated and sEV co-cultured MO3.13 cells. Surprisingly, the protein levels of PCNA tended to enhance in sEV-treated MO3.13 compared to untreated cells after 5 days of culture (Figure 1L). However, this observed increase was not statistically significant (Figure 1M).

### sEV Reduce the Notch Signaling Pathway in MO3.13 Cells

**FIGURE 2:**
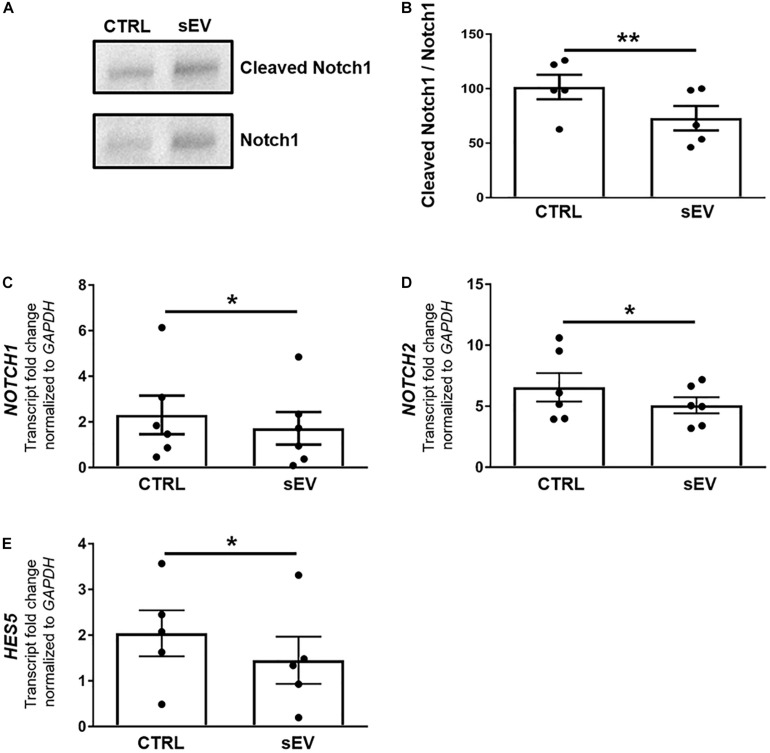
WJ-MSC-derived sEV drive oligodendrocyte maturation in MO3.13 cells by reducing Notch signaling. **(A)** Representative western blot of Notch1 and Cleaved Notch1 (NICD) levels in MO3.13 cells either left untreated or co-cultured with 0.5 μg/mL sEV for 5 days. **(B)** Quantification of the Notch1/NICD ratio (*n* = 5). **(C–E)** Transcription of *NOTCH1* (*n* = 6), *NOTCH2* (*n* = 6), and *HES5* (*n* = 5) by MO3.13 cells either left untreated or co-cultured with 0.5 μg/mL sEV for 5 days. Data are presented as mean ± SEM. *p < 0.05, **p < 0.01.

Notch signaling is well known as a key negative regulator of oligodendroglial maturation (Wang et al., 1998). Therefore, we assessed whether WJ-MSC-derived sEV promote oligodendroglial maturation by inhibiting the activation of Notch pathway. Upon activation, the intracellular membrane-bound Notch transcription factor, known as NICD, gets cleaved, translocates to the nucleus and promotes transcription of Notch target genes. However, sEV significantly reduced the cleavage of NICD in MO3.13 cells compared to untreated cells (*p* = 0.0040) upon 5 days of culture (Figures 2A,B).

Furthermore, sEV significantly decreased the transcription of *NOTCH1* (*p* = 0.0247), *NOTCH2* (*p* = 0.0430), and the Notch target gene *HES5* (*p* = 0.0381) in MO3.13 cells after 5 days of co-culture relative to untreated MO3.13 cells (Figures 2C–E).

### sEV Reduce the MAPK/ERK Signaling Pathway in MO3.13 Cells

**FIGURE 3:**
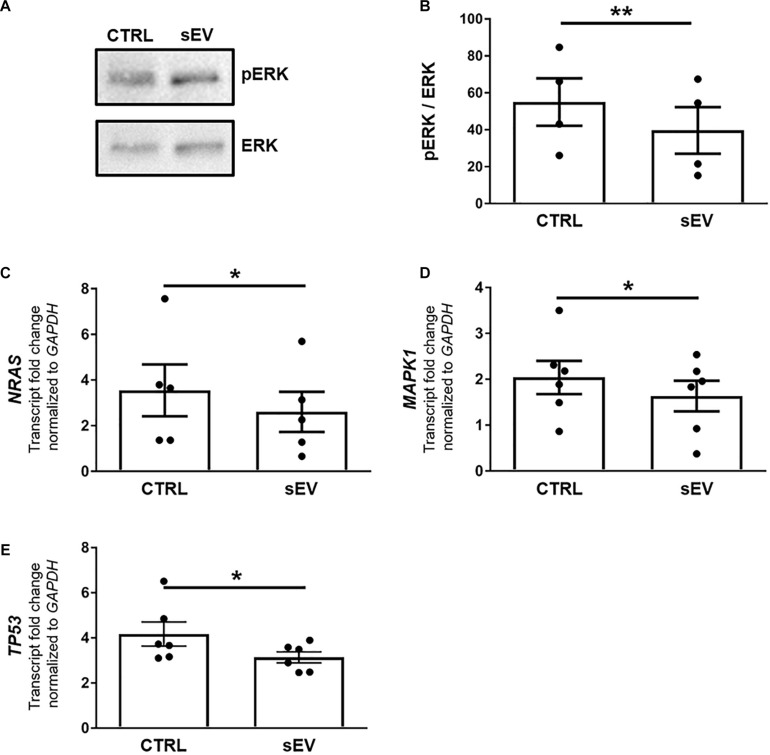
WJ-MSC-derived sEV drive oligodendrocyte maturation in MO3.13 cells by reducing MAPK/ERK signaling. **(A)** Representative western blot of ERK and phosphorylated ERK (pERK) levels in MO3.13 cells either left untreated or co-cultured with 0.5 μg/mL sEV for 5 days. **(B)** Quantification of the ERK/pERK ratio of *n* = 4 replicates. **(C–E)** Transcription of *NRAS* (*n* = 5), *MAPK1* (*n* = 6), and *TP53* (*n* = 6) by MO3.13 cells either left untreated or co-cultured with 0.5 μg/mL sEV for 5 days. Data are presented as mean ± SEM. *p < 0.05, **p < 0.01.

A more recently identified negative regulator of oligodendrocyte differentiation is the MAPK/ERK pathway (Suo et al., 2019; Allan et al., 2021). The MAPK/ERK signaling comprises the activation of RAS, including NRAS, and a subsequent kinase cascade leading, among others, to the phosphorylation of ERK1/2. As shown in Figures 3A,B, the ratio of phosphorylated (p)ERK/ERK was significantly decreased in MO3.13 cells following co-culture with sEV for 5 days relative to untreated cells (*p* = 0.0087). In addition, the transcription of *NRAS* (*p* = 0.0500) and *MAPK1* (*p* = 0.0377), encoding for ERK2, was significantly reduced upon the culture of MO3.13 cells with sEV compared to untreated cells (Figures 3C,D). The gene expression of *TP53* (*p* = 0.0343), encoding for p53, which is known to functionally interact with the MAPK/ERK pathway, was significantly decreased in MO3.13 cells upon co-culture with sEV for 5 days compared to untreated cells (Figure 3E).

### Expression of Mature miR in WJ-MSC and Their Derived sEV

**FIGURE 4:**
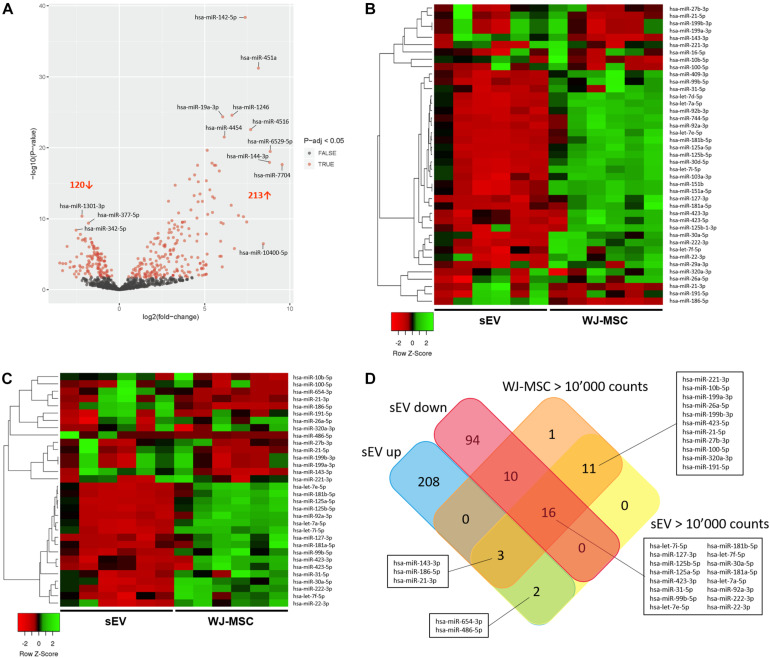
Characterization of miR expressed in WJ-MSC and their derived sEV by Illumina HiSeq Sequencing. **(A)** Volcano plot illustrating miR expression in sEV relative to WJ-MSC. **(B)** Heatmap showing the most prevalent miR expressed in WJ-MSC (mean normalized counts >10,000) relative to sEV. **(C)** Heatmap showing the most prevalent miR expressed in sEV (mean normalized counts >10,000) relative to WJ-MSC. **(D)** Venn plot summarizing the datasets of miR, which were significantly up-regulated in sEV (EV up) compared to WJ-MSC, significantly down-regulated in sEV (EV down) relative to WJ-MSC, and highly abundant in sEV (EV >10,000 counts) and WJ-MSC (WJ-MSC >10,000 counts).

To assess whether Notch and MAPK/ERK pathways in MO3.13 could be regulated by sEV-derived miR, Illumina NextSeq Sequencing was done. WJ-MSC and their derived sEV have been analyzed for the expression of 2,656 known miR. In sEV, 850 miR with mean normalized counts from 1 to more than 10,000 were identified. Thereof, 213 were significantly up-regulated (*p*-adj < 0.05) in WJ-MSC-derived sEV compared to their donor cells, while 120 were down-regulated (*p*-adj < 0.05) (Figure 4A). To identify the most prevalent miR in WJ-MSC and their derived sEV, we set the lower limit of mean normalized counts to 10,000. We found 32 miR with >10,000 mean normalized counts in sEV, and 41 in WJ-MSC (Figures 4B,C).

A Venn diagram was generated using the datasets of the miR that were up-regulated in sEV compared to WJ-MSC (EV up), miR that were down-regulated in sEV compared to WJ-MSC (EV down) and miR that yielded >10,000 mean normalized counts in either sEV (EV >10,000 counts) or WJ-MSC (WJ-MSC >10,000 counts) to identify miR, which were either unique to a certain data set or common to several datasets (Figure 4D). Five of the 213 miR that were up-regulated in sEV relative to their parent cells revealed >10,000 mean normalized counts. Three of these 5 miR, namely miR-143-3p, miR-186-5p, and miR-21-3p, were highly expressed in WJ-MSC as well. Furthermore, 30 miR with >10,000 mean normalized counts were common to WJ-MSC and sEV. In WJ-MSC as well as sEV the miR with the largest mean normalized count was miR-22-3p (WJ-MSC: 517,157.11 counts; sEV: 268,999.81 counts).

For the following KEGG pathway enrichment analysis, miR in sEV with >10,000 mean normalized counts were included.

### WJ-MSC-Derived sEV miR Target Genes Involved in MAPK and Notch Signaling Pathways

**FIGURE 5:**
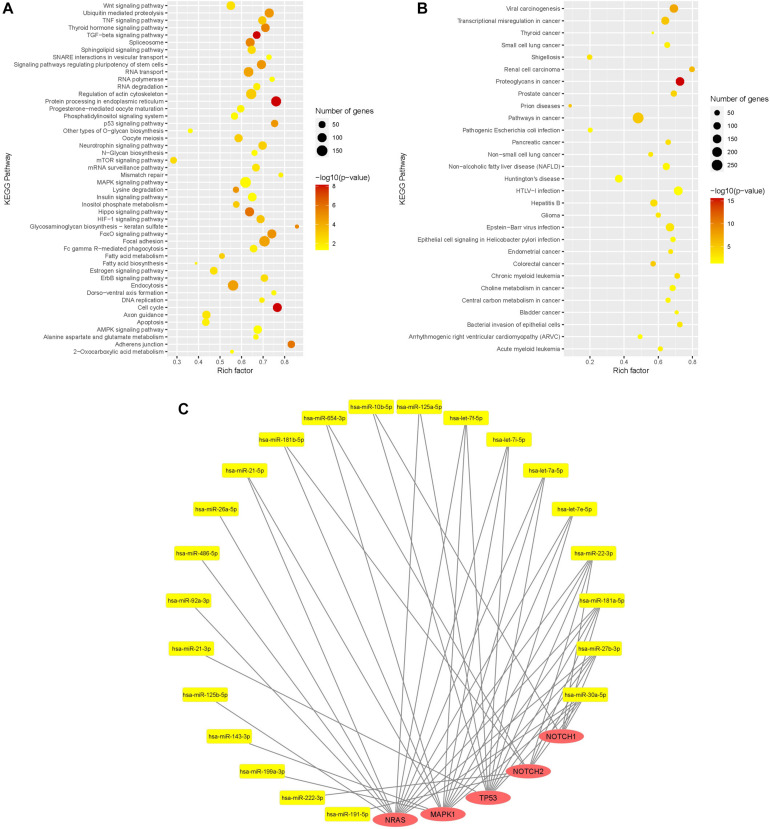
Functional analysis of WJ-MSC-derived sEV miR. **(A,B)** Scatter plot of the Kyoto Encyclopedia of Genes and Genomes (KEGG) pathway enrichment analysis of sEV miR (mean normalized counts >10,000) subdivided into general pathways **(A)** and disease-related pathways **(B)**. **(C)** miR-mRNA interaction network of sEV miRs (yellow) and their target genes (red) being relevant in oligodendrocyte maturation.

KEGG pathway enrichment analysis was done with the 32 most expressed sEV miR (>10,000 mean normalized counts) and revealed 77 significantly enriched signaling pathways, of which 29 pathways were directly assigned to a disease (Figures 5A,B). Among the pathways, which were not specifically attributed to a disease, the keratan sulfate biosynthesis pathway was the pathway with the highest rich factor. Keratan sulfate is a sulfated glycosaminoglycan and known to promote glial scar formation, thereby inhibiting axon regeneration after brain injury (Zhang et al., 2006). Furthermore, the MAPK signaling pathway, as well as the dorso-ventral axis formation pathway, including Notch signaling, were significantly enriched, in which 154 out of 249 genes and 21 out of 28 genes, respectively, were targeted by WJ-MSC-derived sEV miR.

Target genes of WJ-MSC-derived sEV miR were also highly associated with other pathways closely related to premature WMI and oligodendroglial maturation, including the dorso-ventral axis formation pathway, TNF signaling pathway, TGF-beta signaling pathway, p53 signaling pathway, apoptosis, FoxO signaling pathway, Wnt signaling pathway, HIF-1 signaling pathway, neurotrophin signaling pathway, and regulation of actin cytoskeleton (Figure 5A).

Among disease-related signaling cascades, proteoglycans in cancer were revealed to exhibit the highest rich factor (Figure 5B). Further enriched disease-attributed pathways included the signaling cascades ascribed to glioma, Huntington’s disease, prions disease, HTLV-1 infection, and Epstein-Barr virus infection (Figure 5B). HTLV-1 infection and Epstein-Barr virus infection are both associated with demyelinating diseases, namely HTLV-1 associated myelopathy (Osame et al., 1986; Bangham et al., 2015) and multiple sclerosis, respectively (Guan et al., 2019).

The genes encoding for *NRAS* and *MAPK1* (ERK2), two key players of the MAPK/ERK pathway, were targeted by 14 and 13 highly expressed WJ-MSC-sEV miR, respectively (Figure 5C). *TP53*, also being interconnected with the MAPK/ERK signaling pathway, was targeted by 11 WJ-MSC-sEV miR. *NOTCH1* and *NOTCH2* turned out to be predicted targets for 5 and 8 WJ-MSC-sEV miR, respectively.

Mature miR-486-5p and miR-654-3p, which were significantly up-regulated in WJ-MSC-sEV relative to their parental cells, target *NRAS*, and *MAPK1* and *NOTCH2*, respectively.

Furthermore, according to the KEGG pathway enrichment analysis *NRAS*, *MAPK1*, *TP53*, *NOTCH2*, and *NOTCH1* are predicted targets of WJ-MSC-sEV miR-22-3p, miR-181a-5p, miR-27b-3p, and miR-30a-5p. The four let-7 family members (let-7a-5p, let-7e-5p, let-7f-5p, and let-7i-5p) highly expressed in WJ-MSC-sEV were expected to target the 3 MAPK pathway members *NRAS*, *MAPK1*, and *TP53* (Figure 5C).

## Discussion

In fetal brain development, the maturation of the myelin-forming oligodendrocytes is precisely timed to differentiate into mature myelin-generating oligodendrocytes. As myelination starts only in the third trimester of pregnancy, at around week 32 of gestation, preterm birth often leads to injury to immature white matter (Vaes et al., 2019). Most of the cells of the oligodendrocyte lineage in the CNS of neonates born between gestational weeks 24–32 are in the stages of immature OPC and pre-oligodendrocytes (Back et al., 2007; Volpe et al., 2011). Inflammation and hypoxia occurring at preterm birth have been characterized as the main causes of oligodendrocyte progenitor cell injury (Khwaja and Volpe, 2008; Volpe, 2009; Deng, 2010). Cerebral inflammation accompanied by the release of free radicals, glutamate, and pro-inflammatory cytokines (Favrais et al., 2011; Hagberg et al., 2012; van Tilborg et al., 2016), and the absent expression of key anti-oxidant enzymes makes the oligodendrocyte progenitors very vulnerable and leads to their damage (Perrone et al., 2015). Although MSC have been documented to have neuroprotective and –regenerative potential, studies showing direct effects of MSC or their derivatives on oligodendroglial maturation *in vitro* are sparse. Clark and coworkers recently gave evidence that EV isolated from MSC of the chorionic villous tissue drive oligodendroglial maturation, as EV increased the transcription of key mature oligodendrocyte markers in primary murine OPC *in vitro* (Clark et al., 2019). However, studies mostly investigated the impact of MSC on oligodendrocyte specification in neural stem/progenitor cells, rather than on the differentiation of maturation-arrested OPC and pre-oligodendrocytes (Steffenhagen et al., 2012; Oppliger et al., 2017).

Here, we provide solid evidence that human WJ-MSC-derived sEV, isolated by serial centrifugation, physically interacted with the immortalized oligodendrocyte precursor cell line MO3.13 and directly accelerated their maturation. Our study shows that sEV down-regulated the OPC marker PDGFRα in MO3.13 after 5 days of co-culture. Together with the transcription factor Nkx2.2, PDGFRα has been previously shown to be a key inducer of oligodendrocyte differentiation in the developing brain (Zhu et al., 2014). The simultaneous up-regulation of the mature oligodendrocyte marker MBP in sEV-treated MO3.13 cells strongly indicates that the cells matured into premyelinating oligodendrocytes.

Suprisingly, the maturation of MO3.13 cells was not accompanied by a reduced proliferation. There was even a trend to an increased expression of the proliferation marker PCNA in MO3.13 cells after sEV-treatment for 5 days. This observation may be explained by the recent finding of Lei and coworkers that sEV derived from MSC of umbilical cords deliver functional PCNA to their target cells (Lei et al., 2021). They further approved that the deposition of PCNA in target cells is partly responsible for the rejuvenating effects of umbilical cord-derived MSC-sEV.

**FIGURE 6:**
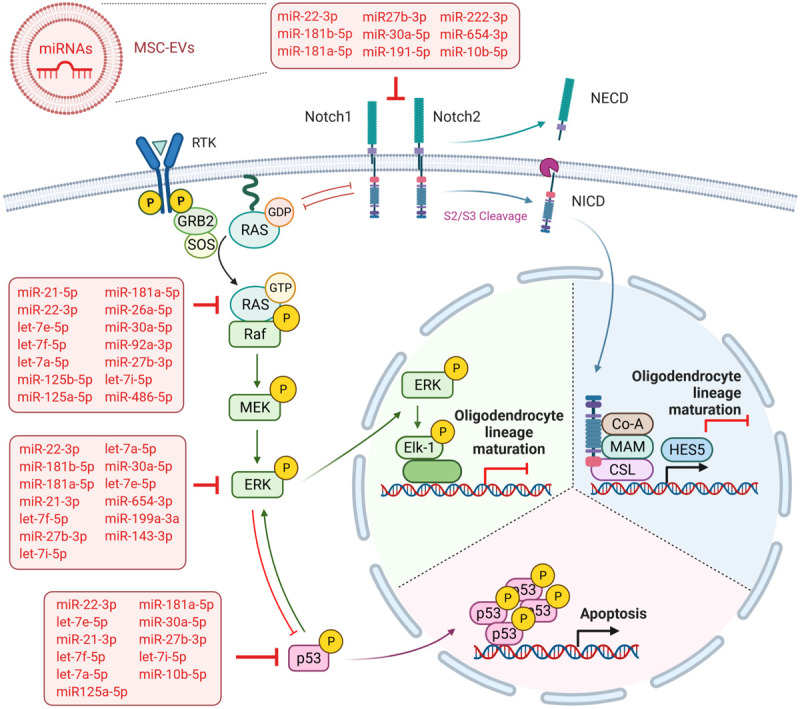
Overview summarizing the predicted targets of WJ-MSC-derived sEV miRs in the Notch and MAPK/ERK signaling cascades. Created with BioRender.com.

The oligodendrocyte specification of neural stem/progenitor cells, as well as their maturation from OPC, are tightly regulated processes based on the activation or inhibition of specific signaling pathways and their intersections. Whereas activated Notch signaling has been identified as an essential step in neural stem/progenitor cells for oligodendrocyte specification (Kagawa et al., 2001), the Notch signaling has to be blocked for the differentiation of OPC into mature oligodendrocytes (Wang et al., 1998). Notch receptor activation, including the intracellular cleavage and nuclear translocation of NICD, primarily blocks OPC differentiation by the transcription of the target gene *HES5* (Liu et al., 2006) (Figure 6). Thereby HES5 associates with the transcription factor SOX10, preventing the binding of SOX10 to the promotor of *MBP* and the induction of its transcription. In the present study, we show that WJ-MSC-derived sEV significantly reduced the cleavage of NICD in the oligodendrocyte precursor cell line MO3.13 after 5 days of co-culture. Furthermore, the transcription of *NOTCH1* and *NOTCH2*, and *HES5* were reduced, corroborating that the sEV-mediated maturation of MO3.13 cells is regulated by the reduced activation of Notch signaling.

A further key pathway negatively regulating OPC maturation is the MAPK/ERK pathway, which has also been shown to intersect with Notch signaling (Sundaram, 2005) (Figure 6). The MAPK/ERK signaling pathway has been previously identified as a main negative regulator of myelination in the demyelinating diseases multiple sclerosis (Suo et al., 2019), the Costello syndrome, and neurofibromatosis type 1, which are Rasopathies caused by germline mutations in genes encoding for members of the MAPK pathway including HRAS (HRas proto-oncogene, GTPase) (Lopez-Juarez et al., 2017; Titus et al., 2017). The multiple sclerosis model has demonstrated that the inhibition of MAPK/ERK cascade was enough to drive OPC differentiation (Suo et al., 2019). In the Costello syndrome and neurofibromatosis type 1 models, it has been shown that besides the blockade of MAPK/ERK pathways, the inhibition of the downstream Notch cascade, either separately or collectively with MAPK/ERK, improved the myelin structure (Lopez-Juarez et al., 2017; Titus et al., 2017). Here, we found that the maturation of MO3.13 cells upon exposure to WJ-MSC-derived sEV was also mediated by reduced ERK phosphorylation and the down-regulated transcription of genes encoding for NRAS and ERK2 (MAPK1). We also recently showed such a WJ-MSC sEV-mediated inhibition of MAPK/ERK signaling in microglial cells in an *in vitro* model of neuroinflammation (Thomi et al., 2019b).

Based on these results and the importance of miR during neuroregeneration in premature WMI (Cho et al., 2019), we performed Illumina HiSeq sequencing, to characterize the miR content of WJ-MSC and WJ-MSC-derived sEV. We detected several highly abundant miR in WJ-MSC and in their derived sEV. Although several miR were expressed to different degrees in sEV and cells, the most abundant miR in both cells and sEV was miR-22-3p. Previously, miR-22-3p has been characterized as being neuroprotective by inhibiting neuronal apoptosis (Jovicic et al., 2013; Ma et al., 2016), which can explain—at least partially–the anti-apoptotic effect of WJ-MSC-derived sEV in neurons recently observed by our group in *in vitro* and *in vivo* models of premature WMI (Joerger-Messerli et al., 2018; Thomi et al., 2019a). In contrast to our study, Fang and coworkers have identified miR-21-5p as the most abundant mature miR in umbilical cord MSC and their derived sEV (Fang et al., 2016). The difference to our results may rely on slightly varying isolation protocols of both cells and sEV. However, miR-21-5p was also highly expressed (>10,000 normalized counts) in our WJ-MSC and sEV, indicating a key function of this miR. It has been well documented that miR-21-5p had neuroprotective impacts by reducing neuronal cell death and promoting angiogenesis (Buller et al., 2010; Ge et al., 2014). Furthermore, it has been published that miR-21-5p in neuron-derived EV inhibited autophagy-mediated nerve injury in an *in vitro* model of traumatic brain injury (Li et al., 2019), elucidating the functional transfer of miR-21-5p from EV to cells. However, as miR regulate the expression of several mRNAs at the same time, and genes might be targeted by different miR, we do not believe that one miR alone is responsible for the beneficial impacts of WJ-MSC and WJ-MSC-derived sEV. Therefore we performed KEGG pathway enrichment analysis to identify a wider spectrum of pathways targeted by the 32 most highly abundant sEV miR (>10,000 normalized counts). Thereby, the analysis revealed that the keratan sulfate biosynthesis was the most enriched pathway. This is of great interest, as the sulfated glycosaminoglycan keratan sulfate is known to trigger glial scar formation, thereby inhibiting axon regeneration after brain injury (Zhang et al., 2006). This led to our hypothesis that targeting this pathway by sEV miR results in reduced formation of glial scars and increased axon regeneration in our premature WMI rat model. The blocking of the keratan biogenesis pathway could explain the reduced glial activation we recently observed in rat pups with premature WMI intranasally treated with WJ-MSC-derived sEV (Thomi et al., 2019b). The impact of WJ-MSC-derived sEV miR on glycosaminoglycans in other diseases was further reflected by the high rich factor of the proteoglycans in the cancer KEGG pathway after the analysis of our high-throughput sequencing results. Glycosaminoglycans have been shown to trigger angiogenesis, invasion and metastasis of cancer cells involving persistent ERK1/2 signaling activation (Yang et al., 2009; Afratis et al., 2012). Several characterized cancer drug candidates have been shown to successfully block the MAPK/ERK pathway (Burotto et al., 2014). The restraining of MAPK/ERK signaling activation could also be a promising therapy approach in premature WMI, as the activation of MAPK/ERK signaling arrests OPC maturation and thereby leads to defective myelination in other demyelinating diseases, as stated before (Titus et al., 2017; Suo et al., 2019). Our high-throughput sequencing results of WJ-MSC-derived sEV miR further strongly indicates that the negative effect of sEV on MAPK/ERK activation in MO3.13 cells may rely on their miR cargo.

In addition, according to our results, the dorso-ventral axis formation pathway, which has a crucial function in neuronal and glial cell development and includes the Notch cascade (Wada et al., 2000; Kagawa et al., 2001), was significantly targeted by sEV miR as well. Interestingly, miR-486-5p and miR-654-3p, which were highly expressed in sEV and significantly up-regulated relative to their parental cells, are predicted to target the genes of key players of the MAPK/ERK and Notch signaling pathways, namely NRAS, and ERK2 (MAPK1) and NOTCH2. This observation additionally supports the advantageous use of WJ-MSC-derived sEV over their maternal cells as a future treatment in premature WMI. However, 22 of the 32 highly expressed sEV miR are predicted to target either the MAPK/ERK pathway *(NRAS, MAPK1)*, the Notch cascade *(NOTCH1, NOTCH2)*, or even both (Figure 6), further accentuating that the sEV miR-mediated regulation of pathways is a rather cooperative acting than the work of an individual miR.

Moreover, KEGG pathway enrichment analysis revealed that WJ-MSC-derived sEV miR target an additional negative regulator of OPC differentiation, namely the Wnt signaling pathway. Active canonical Wnt signaling interferes with oligodendrocyte maturation, thereby slowing down the generation of myelinating oligodendrocytes (Fancy et al., 2009; Feigenson et al., 2009).

As hypo-myelination in premature WMI leads to “secondary” injuries, often affecting demyelinated neurons and axons (Volpe, 2009), future therapy would ideally be multifactorial. As already mentioned before, our group has recently shown that both WJ-MSC and their derived sEV have several beneficial impacts in *in vitro* and *in vivo* models of premature WMI. In particular, we found that the intranasal administration of WJ-MSC prevented myelination deficits and microgliosis in neonatal rats with premature WMI (Oppliger et al., 2016). Most recently, our group could demonstrate that sEV derived from WJ-MSC reduced glial activation, neuronal apoptosis, and rat pup mortality and promoted oligodendrocyte lineage maturation, axon myelination, and learning and memory in young rats suffering from premature WMI after intranasal application (Thomi et al., 2019a,b). Based on our high-throughput sequencing data presented here, we now can provide powerful indications that miR play major roles in these described neuroregenerative effects of WJ-MSC and their derived sEV. For example, we show that the miR of sEV are predicted to significantly target the TNF signaling pathway, which is known to contribute to neuroinflammation. The prognosticated targeting of the p53, apoptosis, and FoxO signaling pathways by sEV miR (Figure 6) are very likely to play a part in the anti-apoptotic action of WJ-MSC-derived sEV we recently observed in neurons (Joerger-Messerli et al., 2018; Thomi et al., 2019a). MAPK signaling functionally interacts with many of the enriched pathways, including p53 and FoxO (Wu, 2004; Asada et al., 2007), highlighting the manifold protective impacts of sEV miR by blocking MAPK/ERK signaling. However, the functionality of the sEV miR have to be further validated in future experiments.

## Conclusion

In conclusion, our study shows that WJ-MSC-derived sEV miR are key regulators of the Notch and ERK/MAPK signaling pathways which allows them to drive the maturation of OPC and pre-oligodendrocytes toward mature myelinating oligodendrocytes (Figure 6). Furthermore, as WJ-MSC-derived sEV miR are predicted to target distinct pathophysiological mechanisms that are involved in premature WMI, we emphasize that miR are key mediators of WJ-MSC-derived sEV beneficial effects in premature WMI.

## Data Availability Statement

The raw data supporting the conclusions of this article will be made available by the authors, without undue reservation, to any qualified researcher.

## Ethics Statement

The studies involving human participants were reviewed and approved by the Ethics Committee of the Canton of Bern. The patients/participants provided their written informed consent to participate in this study.

## Author Contributions

MJ-M, GT, IK, DS, and AS conceived and designed the experiments. MJ-M, GT, IK, PR, and AS analyzed the data. MJ-M, GT, PR, and VH conducted the experiments. MJ-M wrote the manuscript. All authors contributed to the article and approved the submitted version.

## Conflict of Interest

The authors declare that the research was conducted in the absence of any commercial or financial relationships that could be construed as a potential conflict of interest.
